# (*E*)-*N*′-(5-Bromo-2-hy­droxy-3-meth­oxy­benzyl­idene)-1*H*-indole-3-carbo­hydrazide

**DOI:** 10.1107/S1600536811039195

**Published:** 2011-09-30

**Authors:** Xiao-Yan Li

**Affiliations:** a Zibo Vocational Institute, Zibo 255314, People’s Republic of China

## Abstract

There are three independent mol­eculesi n the asymmetric unit of the title compound, C_18_H_16_BrN_3_O_3_, in which the dihedral angles between the indole and benzene rings are 76.9 (2), 4.9 (2), and 70.9 (2)°. All three mol­ecules exist in a *trans* configuration with respect to the methyl­idene units. In each mol­ecule, there is one intra­molecular O—H⋯N hydrogen bond. In the crystal, N—H⋯O hydrogen bonds occur.

## Related literature

For the syntheses and crystal structures of hydrazone compounds, see: Hashemian *et al.* (2011 ▶); Lei (2011 ▶); Shalash *et al.* (2010 ▶). For the crystal structures of similar compounds reported recently by the author, see: Li (2011*a* ▶,*b* ▶).
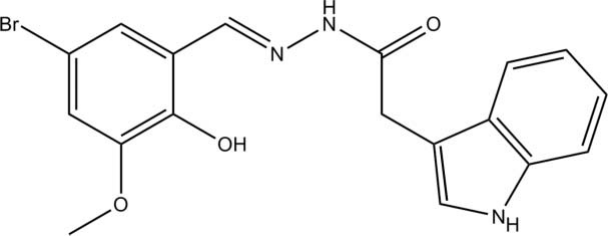


## Experimental

### 

#### Crystal data

C_18_H_16_BrN_3_O_3_
*M* *_r_* = 402.25
Monoclinic, 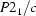

*a* = 15.023 (3) Å
*b* = 13.860 (3) Å
*c* = 24.856 (4) Å
β = 102.192 (2)°
*V* = 5058.8 (17) Å^3^
*Z* = 12
Mo *K*α radiation
μ = 2.46 mm^−1^
*T* = 298 K
0.13 × 0.10 × 0.07 mm

#### Data collection

Bruker SMART CCD area-detector diffractometer
Absorption correction: multi-scan (*SADABS*; Sheldrick, 1996 ▶) *T*_min_ = 0.740, *T*_max_ = 0.847
23383 measured reflections
8466 independent reflections
3662 reflections with *I* > 2σ(*I*)
*R*_int_ = 0.089

#### Refinement

*R*[*F* ^2^ > 2σ(*F* ^2^)] = 0.051
*wR*(*F* ^2^) = 0.125
*S* = 0.97
8466 reflections
700 parameters
24 restraints
H atoms treated by a mixture of independent and constrained refinement
Δρ_max_ = 0.37 e Å^−3^
Δρ_min_ = −0.40 e Å^−3^

### 

Data collection: *SMART* (Bruker, 1998 ▶); cell refinement: *SAINT* (Bruker, 1998 ▶); data reduction: *SAINT*; program(s) used to solve structure: *SHELXS97* (Sheldrick, 2008 ▶); program(s) used to refine structure: *SHELXL97* (Sheldrick, 2008 ▶); molecular graphics: *SHELXTL* (Sheldrick, 2008 ▶); software used to prepare material for publication: *SHELXTL*.

## Supplementary Material

Crystal structure: contains datablock(s) global, I. DOI: 10.1107/S1600536811039195/qm2030sup1.cif

Structure factors: contains datablock(s) I. DOI: 10.1107/S1600536811039195/qm2030Isup2.hkl

Supplementary material file. DOI: 10.1107/S1600536811039195/qm2030Isup3.cml

Additional supplementary materials:  crystallographic information; 3D view; checkCIF report

## Figures and Tables

**Table 1:** Hydrogen-bond geometry (Å, °)

*D*—H⋯*A*	*D*—H	H⋯*A*	*D*⋯*A*	*D*—H⋯*A*
N3—H3⋯O8^i^	0.90 (1)	2.73 (4)	3.453 (8)	138 (5)
N9—H9⋯O2	0.89 (1)	2.16 (2)	3.048 (6)	171 (6)
N6—H6⋯O5^ii^	0.90 (1)	2.28 (2)	3.159 (6)	165 (5)
N2—H2⋯O9^iii^	0.90 (1)	2.01 (2)	2.893 (6)	168 (6)
N5—H5⋯O6^iv^	0.90 (1)	1.99 (1)	2.885 (6)	174 (5)
N8—H8⋯O3^v^	0.90 (1)	1.89 (1)	2.795 (6)	179 (6)
O7—H7⋯N7	0.82	1.90	2.615 (6)	146
O4—H4⋯N4	0.82	1.92	2.634 (6)	145
O1—H1⋯N1	0.82	1.91	2.610 (6)	143

Symmetry codes: (i) 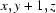; (ii) 
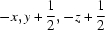
; (iii) 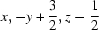; (iv) 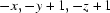; (v) 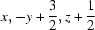.

**Fig. 1.**
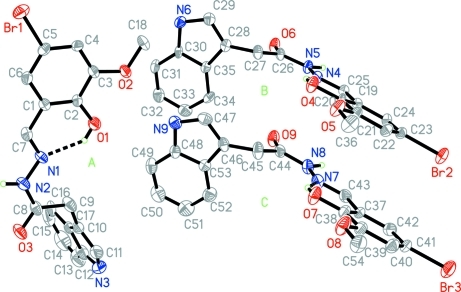
The molecular structure of the three independent molecules of the title compound, showing the atom labelling scheme. The displacement ellipsoids are drawn at the 30% probability level. Intramolecular O—H···N hydrogen bonds are indicated by dashed lines. The C-bound H-atoms have been omitted for clarity.

## supplementary crystallographic information

### Comment

In the last few years, hydrazones have attracted much attention for their syntheses and crystal structures (Hashemian *et al.*, 2011; Lei, 2011; Shalash *et al.*, 2010). As a continuation of our work on such compounds (Li, 2011*a*,*b*), the author reports herein on the crystal structure of the new title hydrazone compound.

In the asymmetric unit of the title compound there are three (A, B and C) independent molecules (Fig. 1). The bond distances and angles are comparable to those observed in similar compounds (Li, 2011*a*,*b*). The dihedral angles between the indole ring and the benzene ring in the three molecules are 76.9 (2), 175.1 (2), and 70.9 (2)° for molecules A, B and C, respectively. All the molecules exist in the *trans* configuration with respect to the methylidene units. In each molecule there is an O—H···N intramolecular hydrogen bond (Table 1).

### Experimental

A mixture of 1*H*-indole-3-carbohydrazide (0.189 g, 1 mmol) and 5-bromo-2-hydroxy-3-methoxybenzaldehyde (0.231 g, 1 mmol) in 30 ml of ethanol containing few drops of acetic acid was refluxed for about 1 h. On cooling to room temperature, a solid precipitate was formed. The solid was filtered and then recrystallized from methanol. Colourless crystals, suitable for X-ray diffraction analysis, were obtained by slow evaporation of a solution of the title compound in methanol.

### Refinement

The NH H-atoms were located from a difference Fourier map and were freely refined. The OH and C-bound H-atoms were positioned geometrically and refined using a riding model: O—H = 0.82 Å, C—H = 0.93 – 0.97 Å, for CH, CH_2_ and CH_3_ H-atoms, with *U*_iso_(H) = k × *U*_eq_(O,*C*), where k = 1.5 for OH and CH_3_ H-atoms, and k = 1.2 for all other H-atoms.

### Crystal data

**Table:**

C_18_H_16_BrN_3_O_3_	*F*(000) = 2448
*M**_r_* = 402.25	*D*_x_ = 1.584 Mg m^−^^3^
Monoclinic, *P*2_1_/*c*	Mo *K*α radiation, λ = 0.71073 Å
*a* = 15.023 (3) Å	Cell parameters from 2672 reflections
*b* = 13.860 (3) Å	θ = 2.2–24.1°
*c* = 24.856 (4) Å	µ = 2.46 mm^−^^1^
β = 102.192 (2)°	*T* = 298 K
*V* = 5058.8 (17) Å^3^	Block, colourless
*Z* = 12	0.13 × 0.10 × 0.07 mm

### Data collection

**Table:**

Bruker SMART CCD area-detector diffractometer	8466 independent reflections
Radiation source: fine-focus sealed tube	3662 reflections with *I* > 2σ(*I*)
graphite	*R*_int_ = 0.089
ω scans	θ_max_ = 25.1°, θ_min_ = 2.1°
Absorption correction: multi-scan (*SADABS*; Sheldrick, 1996)	*h* = −17→17
*T*_min_ = 0.740, *T*_max_ = 0.847	*k* = −16→13
23383 measured reflections	*l* = −29→29

### Refinement

**Table:**

Refinement on *F*^2^	Primary atom site location: structure-invariant direct methods
Least-squares matrix: full	Secondary atom site location: difference Fourier map
*R*[*F*^2^ > 2σ(*F*^2^)] = 0.051	Hydrogen site location: inferred from neighbouring sites
*wR*(*F*^2^) = 0.125	H atoms treated by a mixture of independent and constrained refinement
*S* = 0.97	*w* = 1/[σ^2^(*F*_o_^2^) + (0.0382*P*)^2^] where *P* = (*F*_o_^2^ + 2*F*_c_^2^)/3
8466 reflections	(Δ/σ)_max_ = 0.001
700 parameters	Δρ_max_ = 0.37 e Å^−^^3^
24 restraints	Δρ_min_ = −0.40 e Å^−^^3^

### Special details

**Table:**

Geometry. All e.s.d.'s (except the e.s.d. in the dihedral angle between two l.s. planes) are estimated using the full covariance matrix. The cell e.s.d.'s are taken into account individually in the estimation of e.s.d.'s in distances, angles and torsion angles; correlations between e.s.d.'s in cell parameters are only used when they are defined by crystal symmetry. An approximate (isotropic) treatment of cell e.s.d.'s is used for estimating e.s.d.'s involving l.s. planes.
Refinement. Refinement of *F*^2^ against ALL reflections. The weighted *R*-factor *wR* and goodness of fit *S* are based on *F*^2^, conventional *R*-factors *R* are based on *F*, with *F* set to zero for negative *F*^2^. The threshold expression of *F*^2^ > σ(*F*^2^) is used only for calculating *R*-factors(gt) *etc*. and is not relevant to the choice of reflections for refinement. *R*-factors based on *F*^2^ are statistically about twice as large as those based on *F*, and *R*- factors based on ALL data will be even larger.

### Fractional atomic coordinates and isotropic or equivalent isotropic displacement parameters (Å^2^)

**Table:**

	*x*	*y*	*z*	*U*_iso_*/*U*_eq_	
Br1	−0.13842 (5)	0.56908 (5)	−0.07512 (3)	0.0692 (3)	
Br2	0.45231 (5)	0.10110 (5)	0.56823 (3)	0.0636 (2)	
Br3	0.76185 (5)	0.12527 (6)	0.56406 (3)	0.0737 (3)	
O1	0.1220 (3)	0.7483 (3)	0.11481 (16)	0.0553 (12)	
H1	0.1607	0.7817	0.1049	0.083*	
O2	0.0106 (3)	0.6169 (3)	0.13414 (16)	0.0543 (12)	
O3	0.3570 (3)	1.0251 (3)	0.05230 (17)	0.0708 (14)	
O4	0.1845 (3)	0.2543 (3)	0.37313 (15)	0.0516 (11)	
H4	0.1532	0.2979	0.3816	0.077*	
O5	0.3003 (3)	0.1246 (3)	0.35743 (15)	0.0529 (12)	
O6	−0.0514 (3)	0.5403 (3)	0.43114 (15)	0.0500 (11)	
O7	0.4976 (3)	0.2696 (3)	0.36521 (16)	0.0627 (13)	
H7	0.4652	0.3129	0.3729	0.094*	
O8	0.6141 (3)	0.1372 (3)	0.35256 (17)	0.0634 (13)	
O9	0.2472 (3)	0.5454 (3)	0.41487 (16)	0.0584 (13)	
N1	0.1844 (3)	0.8539 (3)	0.0440 (2)	0.0435 (14)	
N2	0.2421 (4)	0.9215 (4)	0.0291 (2)	0.0528 (15)	
N3	0.4718 (5)	1.0844 (7)	0.2278 (3)	0.094 (3)	
N4	0.1192 (3)	0.3660 (3)	0.44138 (19)	0.0381 (13)	
N5	0.0606 (3)	0.4335 (3)	0.45502 (19)	0.0395 (13)	
N6	−0.1466 (3)	0.6116 (4)	0.25086 (19)	0.0441 (13)	
N7	0.4287 (4)	0.3826 (3)	0.4301 (2)	0.0470 (14)	
N8	0.3669 (4)	0.4477 (4)	0.4423 (2)	0.0488 (14)	
N9	0.1693 (3)	0.6162 (4)	0.2334 (2)	0.0485 (14)	
C1	0.0654 (4)	0.7401 (4)	0.0170 (3)	0.0388 (16)	
C2	0.0651 (4)	0.7112 (4)	0.0700 (2)	0.0357 (15)	
C3	0.0035 (4)	0.6407 (4)	0.0800 (3)	0.0425 (16)	
C4	−0.0565 (4)	0.5993 (4)	0.0363 (2)	0.0419 (16)	
H4A	−0.0972	0.5523	0.0426	0.050*	
C5	−0.0556 (4)	0.6283 (4)	−0.0165 (2)	0.0423 (16)	
C6	0.0027 (4)	0.6985 (4)	−0.0275 (2)	0.0440 (16)	
H6A	0.0009	0.7182	−0.0634	0.053*	
C7	0.1276 (4)	0.8133 (4)	0.0048 (3)	0.0451 (17)	
H7A	0.1263	0.8307	−0.0315	0.054*	
C8	0.3041 (5)	0.9683 (5)	0.0672 (3)	0.0513 (19)	
C9	0.3046 (5)	0.9496 (4)	0.1269 (3)	0.067 (2)	
H9A	0.2424	0.9515	0.1320	0.080*	
H9B	0.3280	0.8852	0.1362	0.080*	
C10	0.3597 (5)	1.0191 (5)	0.1653 (3)	0.0482 (17)	
C11	0.4412 (6)	1.0053 (6)	0.1978 (4)	0.082 (3)	
H11	0.4736	0.9477	0.1996	0.098*	
C12	0.4076 (7)	1.1530 (6)	0.2146 (3)	0.069 (2)	
C13	0.4077 (7)	1.2471 (7)	0.2351 (3)	0.106 (3)	
H13	0.4569	1.2724	0.2602	0.128*	
C14	0.3348 (9)	1.2962 (7)	0.2166 (4)	0.117 (4)	
H14	0.3336	1.3581	0.2309	0.141*	
C15	0.2586 (7)	1.2692 (7)	0.1785 (4)	0.107 (3)	
H15	0.2095	1.3108	0.1677	0.129*	
C16	0.2583 (6)	1.1721 (6)	0.1558 (3)	0.077 (2)	
H16	0.2095	1.1488	0.1296	0.093*	
C17	0.3359 (5)	1.1157 (5)	0.1759 (3)	0.0471 (17)	
C18	−0.0485 (4)	0.5440 (4)	0.1464 (2)	0.0617 (19)	
H18A	−0.1104	0.5603	0.1299	0.093*	
H18B	−0.0419	0.5388	0.1855	0.093*	
H18C	−0.0333	0.4834	0.1319	0.093*	
C19	0.2422 (4)	0.2557 (4)	0.4718 (2)	0.0341 (15)	
C20	0.2423 (4)	0.2217 (4)	0.4187 (2)	0.0352 (15)	
C21	0.3056 (4)	0.1522 (4)	0.4109 (2)	0.0408 (16)	
C22	0.3683 (4)	0.1168 (4)	0.4554 (2)	0.0394 (15)	
H22	0.4110	0.0710	0.4503	0.047*	
C23	0.3665 (4)	0.1504 (4)	0.5075 (2)	0.0370 (15)	
C24	0.3055 (4)	0.2189 (4)	0.5165 (2)	0.0400 (16)	
H24	0.3062	0.2406	0.5519	0.048*	
C25	0.1756 (4)	0.3268 (4)	0.4806 (2)	0.0377 (16)	
H25	0.1741	0.3443	0.5165	0.045*	
C26	0.0009 (4)	0.4826 (4)	0.4160 (2)	0.0372 (16)	
C27	0.0038 (4)	0.4648 (4)	0.3569 (2)	0.0438 (16)	
H27A	0.0670	0.4630	0.3535	0.053*	
H27B	−0.0225	0.4020	0.3463	0.053*	
C28	−0.0453 (4)	0.5385 (4)	0.3178 (2)	0.0381 (16)	
C29	−0.1248 (5)	0.5292 (4)	0.2810 (3)	0.0459 (17)	
H29	−0.1604	0.4737	0.2767	0.055*	
C30	−0.0782 (4)	0.6774 (4)	0.2689 (2)	0.0362 (15)	
C31	−0.0676 (5)	0.7714 (4)	0.2514 (2)	0.0493 (17)	
H31	−0.1119	0.8001	0.2244	0.059*	
C32	0.0090 (5)	0.8194 (4)	0.2748 (3)	0.058 (2)	
H32	0.0181	0.8815	0.2629	0.070*	
C33	0.0748 (5)	0.7777 (5)	0.3165 (3)	0.0570 (19)	
H33	0.1260	0.8134	0.3325	0.068*	
C34	0.0656 (4)	0.6847 (4)	0.3345 (2)	0.0441 (17)	
H34	0.1103	0.6566	0.3615	0.053*	
C35	−0.0130 (4)	0.6344 (4)	0.3106 (2)	0.0331 (15)	
C36	0.3667 (4)	0.0585 (4)	0.3462 (2)	0.0610 (19)	
H36A	0.4263	0.0857	0.3581	0.091*	
H36B	0.3556	0.0460	0.3073	0.091*	
H36C	0.3628	−0.0008	0.3655	0.091*	
C37	0.5529 (4)	0.2773 (4)	0.4631 (3)	0.0392 (16)	
C38	0.5555 (4)	0.2401 (5)	0.4113 (3)	0.0473 (17)	
C39	0.6188 (4)	0.1688 (4)	0.4054 (3)	0.0479 (17)	
C40	0.6801 (4)	0.1356 (4)	0.4512 (3)	0.0517 (18)	
H40	0.7223	0.0882	0.4476	0.062*	
C41	0.6779 (4)	0.1733 (4)	0.5021 (3)	0.0468 (17)	
C42	0.6149 (4)	0.2428 (4)	0.5089 (3)	0.0478 (17)	
H42	0.6140	0.2663	0.5439	0.057*	
C43	0.4858 (4)	0.3477 (4)	0.4714 (3)	0.0487 (18)	
H43	0.4841	0.3678	0.5069	0.058*	
C44	0.3035 (5)	0.4900 (4)	0.4022 (3)	0.0478 (18)	
C45	0.3064 (4)	0.4670 (4)	0.3435 (2)	0.0585 (19)	
H45A	0.3695	0.4600	0.3406	0.070*	
H45B	0.2765	0.4055	0.3337	0.070*	
C46	0.2620 (5)	0.5416 (4)	0.3027 (3)	0.0456 (17)	
C47	0.1861 (5)	0.5327 (5)	0.2626 (3)	0.0521 (18)	
H47	0.1505	0.4773	0.2559	0.063*	
C48	0.2355 (4)	0.6824 (5)	0.2549 (3)	0.0413 (16)	
C49	0.2486 (5)	0.7766 (5)	0.2387 (3)	0.060 (2)	
H49	0.2088	0.8058	0.2095	0.072*	
C50	0.3221 (6)	0.8232 (5)	0.2678 (3)	0.072 (2)	
H50	0.3324	0.8863	0.2579	0.087*	
C51	0.3831 (5)	0.7821 (6)	0.3117 (3)	0.077 (2)	
H51	0.4327	0.8173	0.3305	0.093*	
C52	0.3694 (5)	0.6884 (5)	0.3271 (3)	0.065 (2)	
H52	0.4100	0.6599	0.3562	0.078*	
C53	0.2944 (4)	0.6371 (4)	0.2989 (3)	0.0413 (16)	
C54	0.6771 (4)	0.0682 (4)	0.3442 (2)	0.064 (2)	
H54A	0.7375	0.0941	0.3550	0.096*	
H54B	0.6655	0.0508	0.3059	0.096*	
H54C	0.6717	0.0120	0.3659	0.096*	
H8	0.363 (4)	0.456 (4)	0.4777 (8)	0.080*	
H5	0.060 (4)	0.437 (4)	0.4910 (7)	0.080*	
H2	0.240 (4)	0.924 (4)	−0.0074 (7)	0.080*	
H6	−0.196 (2)	0.622 (4)	0.2240 (17)	0.080*	
H9	0.119 (2)	0.620 (4)	0.2067 (17)	0.080*	
H3	0.5281 (18)	1.084 (4)	0.249 (2)	0.080*	

### Atomic displacement parameters (Å^2^)

**Table:**

	*U*^11^	*U*^22^	*U*^33^	*U*^12^	*U*^13^	*U*^23^
Br1	0.0570 (5)	0.0826 (5)	0.0592 (5)	−0.0098 (4)	−0.0077 (4)	−0.0082 (4)
Br2	0.0562 (5)	0.0761 (5)	0.0501 (4)	0.0204 (4)	−0.0076 (4)	0.0116 (4)
Br3	0.0615 (5)	0.0934 (6)	0.0574 (5)	0.0157 (5)	−0.0074 (4)	0.0110 (4)
O1	0.066 (3)	0.062 (3)	0.038 (3)	−0.019 (3)	0.010 (2)	−0.003 (2)
O2	0.065 (3)	0.054 (3)	0.043 (3)	−0.021 (2)	0.009 (2)	0.010 (2)
O3	0.099 (4)	0.069 (3)	0.050 (3)	−0.045 (3)	0.027 (3)	−0.007 (2)
O4	0.057 (3)	0.059 (3)	0.037 (3)	0.025 (2)	0.006 (2)	0.002 (2)
O5	0.050 (3)	0.069 (3)	0.037 (3)	0.024 (2)	0.003 (2)	−0.011 (2)
O6	0.061 (3)	0.051 (3)	0.040 (3)	0.031 (2)	0.016 (2)	0.006 (2)
O7	0.063 (3)	0.077 (4)	0.046 (3)	0.035 (3)	0.008 (3)	0.011 (3)
O8	0.061 (3)	0.082 (3)	0.046 (3)	0.036 (3)	0.009 (3)	0.000 (3)
O9	0.068 (3)	0.060 (3)	0.051 (3)	0.032 (3)	0.020 (3)	0.011 (2)
N1	0.049 (4)	0.035 (3)	0.052 (4)	−0.009 (3)	0.022 (3)	0.001 (3)
N2	0.059 (4)	0.052 (4)	0.052 (4)	−0.014 (3)	0.022 (4)	−0.003 (3)
N3	0.044 (5)	0.146 (8)	0.076 (6)	−0.031 (6)	−0.023 (4)	0.032 (5)
N4	0.037 (3)	0.028 (3)	0.050 (3)	0.014 (3)	0.011 (3)	0.007 (3)
N5	0.046 (3)	0.040 (3)	0.032 (3)	0.015 (3)	0.007 (3)	0.003 (3)
N6	0.043 (4)	0.045 (3)	0.036 (3)	0.005 (3)	−0.009 (3)	−0.004 (3)
N7	0.055 (4)	0.041 (3)	0.050 (4)	0.011 (3)	0.022 (3)	0.004 (3)
N8	0.059 (4)	0.049 (3)	0.044 (4)	0.016 (3)	0.022 (3)	0.006 (3)
N9	0.049 (4)	0.051 (4)	0.042 (4)	−0.003 (4)	0.001 (3)	−0.002 (3)
C1	0.040 (4)	0.036 (4)	0.043 (4)	0.004 (3)	0.016 (4)	0.001 (3)
C2	0.045 (4)	0.030 (4)	0.032 (4)	−0.001 (3)	0.008 (4)	−0.001 (3)
C3	0.047 (4)	0.039 (4)	0.045 (4)	0.001 (4)	0.017 (4)	0.008 (3)
C4	0.038 (4)	0.038 (4)	0.046 (4)	−0.006 (3)	0.001 (4)	0.004 (3)
C5	0.042 (4)	0.047 (4)	0.035 (4)	0.001 (3)	0.000 (3)	−0.009 (3)
C6	0.050 (4)	0.046 (4)	0.035 (4)	0.001 (4)	0.006 (4)	0.003 (3)
C7	0.060 (5)	0.039 (4)	0.039 (4)	−0.001 (4)	0.016 (4)	0.000 (3)
C8	0.074 (6)	0.037 (4)	0.048 (5)	−0.008 (4)	0.023 (4)	−0.003 (4)
C9	0.096 (6)	0.058 (5)	0.053 (5)	−0.027 (4)	0.032 (4)	−0.013 (4)
C10	0.047 (5)	0.056 (5)	0.042 (4)	−0.008 (4)	0.010 (4)	0.002 (4)
C11	0.066 (7)	0.086 (7)	0.093 (7)	−0.001 (6)	0.020 (6)	0.033 (6)
C12	0.089 (7)	0.065 (6)	0.049 (5)	−0.037 (5)	0.010 (5)	−0.005 (5)
C13	0.147 (7)	0.111 (7)	0.063 (5)	−0.048 (6)	0.026 (5)	−0.012 (5)
C14	0.178 (8)	0.083 (6)	0.107 (7)	−0.011 (6)	0.070 (7)	−0.010 (5)
C15	0.126 (7)	0.103 (6)	0.107 (7)	0.042 (6)	0.056 (6)	0.057 (6)
C16	0.092 (7)	0.095 (7)	0.047 (5)	0.004 (6)	0.022 (5)	0.034 (5)
C17	0.058 (5)	0.048 (5)	0.035 (4)	−0.016 (4)	0.008 (4)	0.003 (4)
C18	0.072 (5)	0.060 (4)	0.058 (5)	−0.012 (4)	0.024 (4)	0.020 (4)
C19	0.040 (4)	0.030 (3)	0.032 (4)	0.002 (3)	0.007 (3)	0.002 (3)
C20	0.039 (4)	0.031 (4)	0.034 (4)	0.016 (3)	0.003 (4)	0.011 (3)
C21	0.041 (4)	0.048 (4)	0.030 (4)	0.004 (3)	0.000 (3)	−0.004 (3)
C22	0.035 (4)	0.037 (4)	0.045 (4)	0.008 (3)	0.007 (3)	−0.005 (3)
C23	0.032 (4)	0.036 (4)	0.042 (4)	0.006 (3)	0.003 (3)	0.001 (3)
C24	0.048 (4)	0.039 (4)	0.031 (4)	0.005 (3)	0.005 (3)	0.006 (3)
C25	0.038 (4)	0.038 (4)	0.035 (4)	0.002 (3)	0.006 (3)	0.002 (3)
C26	0.049 (4)	0.028 (4)	0.036 (4)	0.009 (3)	0.011 (4)	0.005 (3)
C27	0.058 (4)	0.034 (4)	0.041 (4)	0.013 (3)	0.013 (4)	0.006 (3)
C28	0.041 (4)	0.042 (4)	0.031 (4)	0.013 (4)	0.008 (3)	−0.004 (3)
C29	0.062 (5)	0.029 (4)	0.047 (4)	0.002 (4)	0.011 (4)	−0.001 (3)
C30	0.053 (5)	0.033 (4)	0.021 (4)	0.001 (3)	0.002 (3)	0.000 (3)
C31	0.067 (5)	0.040 (4)	0.035 (4)	0.004 (4)	−0.001 (4)	0.006 (3)
C32	0.086 (6)	0.038 (4)	0.049 (5)	−0.004 (4)	0.012 (5)	−0.001 (4)
C33	0.064 (5)	0.055 (5)	0.051 (5)	−0.018 (4)	0.010 (4)	−0.017 (4)
C34	0.039 (4)	0.057 (5)	0.030 (4)	0.004 (4)	−0.006 (3)	−0.009 (3)
C35	0.044 (4)	0.032 (4)	0.021 (4)	0.004 (3)	0.003 (3)	−0.009 (3)
C36	0.060 (5)	0.070 (5)	0.054 (5)	0.018 (4)	0.014 (4)	−0.019 (4)
C37	0.029 (4)	0.045 (4)	0.041 (4)	0.006 (3)	0.003 (3)	0.009 (3)
C38	0.039 (4)	0.058 (5)	0.043 (5)	0.006 (4)	0.004 (4)	0.009 (4)
C39	0.040 (4)	0.060 (5)	0.043 (5)	0.004 (4)	0.009 (4)	0.007 (4)
C40	0.045 (4)	0.055 (4)	0.055 (5)	0.012 (4)	0.011 (4)	0.005 (4)
C41	0.034 (4)	0.054 (4)	0.047 (5)	0.003 (4)	−0.005 (4)	0.014 (4)
C42	0.047 (5)	0.055 (4)	0.042 (4)	−0.004 (4)	0.011 (4)	−0.004 (4)
C43	0.051 (5)	0.047 (4)	0.052 (5)	0.005 (4)	0.023 (4)	0.004 (4)
C44	0.050 (5)	0.039 (4)	0.056 (5)	0.007 (4)	0.015 (4)	0.014 (4)
C45	0.078 (5)	0.051 (4)	0.050 (5)	0.024 (4)	0.022 (4)	0.012 (4)
C46	0.061 (5)	0.042 (4)	0.038 (4)	0.009 (4)	0.019 (4)	0.003 (4)
C47	0.060 (5)	0.038 (4)	0.060 (5)	−0.007 (4)	0.017 (4)	−0.003 (4)
C48	0.046 (5)	0.044 (4)	0.034 (4)	0.005 (4)	0.010 (4)	−0.002 (4)
C49	0.091 (6)	0.041 (5)	0.047 (5)	0.008 (4)	0.013 (5)	0.004 (4)
C50	0.109 (7)	0.057 (5)	0.054 (6)	−0.019 (5)	0.024 (5)	−0.019 (5)
C51	0.094 (7)	0.079 (6)	0.054 (5)	−0.028 (5)	0.002 (5)	−0.029 (5)
C52	0.071 (6)	0.064 (5)	0.056 (5)	−0.002 (5)	0.005 (4)	−0.006 (4)
C53	0.045 (5)	0.039 (4)	0.040 (4)	0.001 (4)	0.009 (4)	−0.005 (3)
C54	0.062 (5)	0.079 (5)	0.056 (5)	0.021 (4)	0.022 (4)	0.001 (4)

### Geometric parameters (Å, °)

**Table:**

Br1—C5	1.892 (6)	C16—H16	0.9300
Br2—C23	1.893 (5)	C18—H18A	0.9600
Br3—C41	1.894 (6)	C18—H18B	0.9600
O1—C2	1.354 (6)	C18—H18C	0.9600
O1—H1	0.8200	C19—C24	1.396 (7)
O2—C3	1.368 (6)	C19—C20	1.402 (7)
O2—C18	1.421 (6)	C19—C25	1.454 (7)
O3—C8	1.231 (6)	C20—C21	1.397 (7)
O4—C20	1.350 (6)	C21—C22	1.382 (7)
O4—H4	0.8200	C22—C23	1.382 (7)
O5—C21	1.369 (6)	C22—H22	0.9300
O5—C36	1.425 (6)	C23—C24	1.370 (7)
O6—C26	1.234 (6)	C24—H24	0.9300
O7—C38	1.346 (6)	C25—H25	0.9300
O7—H7	0.8200	C26—C27	1.500 (7)
O8—C39	1.371 (7)	C27—C28	1.493 (7)
O8—C54	1.392 (6)	C27—H27A	0.9700
O9—C44	1.230 (6)	C27—H27B	0.9700
N1—C7	1.281 (6)	C28—C29	1.348 (7)
N1—N2	1.380 (6)	C28—C35	1.439 (7)
N2—C8	1.346 (7)	C29—H29	0.9300
N2—H2	0.901 (10)	C30—C31	1.393 (7)
N3—C12	1.344 (9)	C30—C35	1.400 (7)
N3—C11	1.352 (9)	C31—C32	1.349 (7)
N3—H3	0.896 (10)	C31—H31	0.9300
N4—C25	1.271 (6)	C32—C33	1.398 (8)
N4—N5	1.376 (6)	C32—H32	0.9300
N5—C26	1.356 (6)	C33—C34	1.381 (7)
N5—H5	0.898 (10)	C33—H33	0.9300
N6—C29	1.367 (7)	C34—C35	1.391 (7)
N6—C30	1.376 (7)	C34—H34	0.9300
N6—H6	0.900 (10)	C36—H36A	0.9600
N7—C43	1.285 (7)	C36—H36B	0.9600
N7—N8	1.373 (6)	C36—H36C	0.9600
N8—C44	1.357 (7)	C37—C42	1.394 (7)
N8—H8	0.902 (10)	C37—C38	1.397 (8)
N9—C47	1.361 (7)	C37—C43	1.448 (7)
N9—C48	1.376 (7)	C38—C39	1.401 (7)
N9—H9	0.894 (10)	C39—C40	1.383 (7)
C1—C2	1.380 (7)	C40—C41	1.376 (8)
C1—C6	1.415 (7)	C40—H40	0.9300
C1—C7	1.454 (7)	C41—C42	1.386 (7)
C2—C3	1.403 (7)	C42—H42	0.9300
C3—C4	1.381 (7)	C43—H43	0.9300
C4—C5	1.377 (7)	C44—C45	1.505 (8)
C4—H4A	0.9300	C45—C46	1.502 (7)
C5—C6	1.373 (7)	C45—H45A	0.9700
C6—H6A	0.9300	C45—H45B	0.9700
C7—H7A	0.9300	C46—C47	1.352 (7)
C8—C9	1.504 (8)	C46—C53	1.420 (8)
C9—C10	1.479 (8)	C47—H47	0.9300
C9—H9A	0.9700	C48—C49	1.393 (8)
C9—H9B	0.9700	C48—C53	1.401 (7)
C10—C11	1.331 (9)	C49—C50	1.350 (8)
C10—C17	1.425 (8)	C49—H49	0.9300
C11—H11	0.9300	C50—C51	1.390 (9)
C12—C17	1.383 (9)	C50—H50	0.9300
C12—C13	1.401 (10)	C51—C52	1.381 (8)
C13—C14	1.289 (11)	C51—H51	0.9300
C13—H13	0.9300	C52—C53	1.391 (8)
C14—C15	1.375 (11)	C52—H52	0.9300
C14—H14	0.9300	C54—H54A	0.9600
C15—C16	1.458 (10)	C54—H54B	0.9600
C15—H15	0.9300	C54—H54C	0.9600
C16—C17	1.405 (8)		
			
C2—O1—H1	109.5	C19—C24—H24	120.3
C3—O2—C18	117.4 (5)	N4—C25—C19	122.8 (6)
C20—O4—H4	109.5	N4—C25—H25	118.6
C21—O5—C36	118.0 (4)	C19—C25—H25	118.6
C38—O7—H7	109.5	O6—C26—N5	118.3 (5)
C39—O8—C54	117.6 (5)	O6—C26—C27	123.8 (6)
C7—N1—N2	116.6 (5)	N5—C26—C27	117.8 (5)
C8—N2—N1	121.2 (5)	C28—C27—C26	114.4 (5)
C8—N2—H2	124 (4)	C28—C27—H27A	108.7
N1—N2—H2	114 (4)	C26—C27—H27A	108.7
C12—N3—C11	107.2 (7)	C28—C27—H27B	108.7
C12—N3—H3	134 (4)	C26—C27—H27B	108.7
C11—N3—H3	119 (4)	H27A—C27—H27B	107.6
C25—N4—N5	117.3 (5)	C29—C28—C35	106.1 (6)
C26—N5—N4	121.7 (5)	C29—C28—C27	128.4 (6)
C26—N5—H5	122 (4)	C35—C28—C27	125.4 (6)
N4—N5—H5	115 (4)	C28—C29—N6	111.6 (6)
C29—N6—C30	107.3 (5)	C28—C29—H29	124.2
C29—N6—H6	128 (4)	N6—C29—H29	124.2
C30—N6—H6	125 (4)	N6—C30—C31	130.1 (6)
C43—N7—N8	116.1 (5)	N6—C30—C35	108.5 (5)
C44—N8—N7	121.7 (5)	C31—C30—C35	121.4 (6)
C44—N8—H8	119 (4)	C32—C31—C30	118.1 (6)
N7—N8—H8	119 (4)	C32—C31—H31	121.0
C47—N9—C48	108.8 (5)	C30—C31—H31	121.0
C47—N9—H9	118 (4)	C31—C32—C33	121.4 (6)
C48—N9—H9	133 (4)	C31—C32—H32	119.3
C2—C1—C6	119.4 (5)	C33—C32—H32	119.3
C2—C1—C7	122.3 (6)	C34—C33—C32	121.5 (6)
C6—C1—C7	118.3 (6)	C34—C33—H33	119.3
O1—C2—C1	123.1 (5)	C32—C33—H33	119.3
O1—C2—C3	116.4 (5)	C33—C34—C35	117.6 (6)
C1—C2—C3	120.4 (6)	C33—C34—H34	121.2
O2—C3—C4	125.1 (5)	C35—C34—H34	121.2
O2—C3—C2	115.1 (6)	C34—C35—C30	120.0 (5)
C4—C3—C2	119.8 (6)	C34—C35—C28	133.4 (6)
C5—C4—C3	119.5 (5)	C30—C35—C28	106.5 (5)
C5—C4—H4A	120.3	O5—C36—H36A	109.5
C3—C4—H4A	120.3	O5—C36—H36B	109.5
C6—C5—C4	122.1 (6)	H36A—C36—H36B	109.5
C6—C5—Br1	119.8 (5)	O5—C36—H36C	109.5
C4—C5—Br1	118.1 (5)	H36A—C36—H36C	109.5
C5—C6—C1	118.8 (6)	H36B—C36—H36C	109.5
C5—C6—H6A	120.6	C42—C37—C38	118.9 (6)
C1—C6—H6A	120.6	C42—C37—C43	118.7 (6)
N1—C7—C1	120.1 (6)	C38—C37—C43	122.4 (6)
N1—C7—H7A	120.0	O7—C38—C37	122.3 (6)
C1—C7—H7A	120.0	O7—C38—C39	117.1 (6)
O3—C8—N2	119.4 (6)	C37—C38—C39	120.6 (6)
O3—C8—C9	122.5 (7)	O8—C39—C40	124.9 (6)
N2—C8—C9	118.1 (6)	O8—C39—C38	115.2 (6)
C10—C9—C8	114.3 (5)	C40—C39—C38	119.8 (6)
C10—C9—H9A	108.7	C41—C40—C39	119.3 (6)
C8—C9—H9A	108.7	C41—C40—H40	120.3
C10—C9—H9B	108.7	C39—C40—H40	120.3
C8—C9—H9B	108.7	C40—C41—C42	121.8 (6)
H9A—C9—H9B	107.6	C40—C41—Br3	118.3 (5)
C11—C10—C17	104.9 (7)	C42—C41—Br3	119.9 (5)
C11—C10—C9	128.0 (8)	C41—C42—C37	119.6 (6)
C17—C10—C9	127.1 (7)	C41—C42—H42	120.2
C10—C11—N3	112.3 (8)	C37—C42—H42	120.2
C10—C11—H11	123.8	N7—C43—C37	120.5 (6)
N3—C11—H11	123.8	N7—C43—H43	119.7
N3—C12—C17	108.6 (7)	C37—C43—H43	119.7
N3—C12—C13	128.6 (10)	O9—C44—N8	119.8 (6)
C17—C12—C13	122.8 (9)	O9—C44—C45	122.8 (6)
C14—C13—C12	115.3 (10)	N8—C44—C45	117.4 (6)
C14—C13—H13	122.4	C46—C45—C44	114.1 (5)
C12—C13—H13	122.4	C46—C45—H45A	108.7
C13—C14—C15	128.5 (11)	C44—C45—H45A	108.7
C13—C14—H14	115.7	C46—C45—H45B	108.7
C15—C14—H14	115.7	C44—C45—H45B	108.7
C14—C15—C16	116.9 (9)	H45A—C45—H45B	107.6
C14—C15—H15	121.6	C47—C46—C53	106.4 (6)
C16—C15—H15	121.6	C47—C46—C45	128.3 (6)
C17—C16—C15	116.2 (8)	C53—C46—C45	125.3 (7)
C17—C16—H16	121.9	C46—C47—N9	110.4 (6)
C15—C16—H16	121.9	C46—C47—H47	124.8
C12—C17—C16	120.3 (7)	N9—C47—H47	124.8
C12—C17—C10	106.9 (7)	N9—C48—C49	130.4 (7)
C16—C17—C10	132.8 (7)	N9—C48—C53	106.8 (5)
O2—C18—H18A	109.5	C49—C48—C53	122.8 (7)
O2—C18—H18B	109.5	C50—C49—C48	116.3 (7)
H18A—C18—H18B	109.5	C50—C49—H49	121.9
O2—C18—H18C	109.5	C48—C49—H49	121.9
H18A—C18—H18C	109.5	C49—C50—C51	123.6 (7)
H18B—C18—H18C	109.5	C49—C50—H50	118.2
C24—C19—C20	119.4 (5)	C51—C50—H50	118.2
C24—C19—C25	120.2 (5)	C52—C51—C50	119.4 (7)
C20—C19—C25	120.5 (5)	C52—C51—H51	120.3
O4—C20—C21	116.7 (5)	C50—C51—H51	120.3
O4—C20—C19	123.3 (5)	C51—C52—C53	119.6 (7)
C21—C20—C19	119.9 (5)	C51—C52—H52	120.2
O5—C21—C22	124.7 (5)	C53—C52—H52	120.2
O5—C21—C20	115.2 (5)	C52—C53—C48	118.4 (6)
C22—C21—C20	120.1 (6)	C52—C53—C46	134.1 (7)
C21—C22—C23	119.1 (5)	C48—C53—C46	107.5 (6)
C21—C22—H22	120.4	O8—C54—H54A	109.5
C23—C22—H22	120.4	O8—C54—H54B	109.5
C24—C23—C22	122.1 (5)	H54A—C54—H54B	109.5
C24—C23—Br2	119.2 (5)	O8—C54—H54C	109.5
C22—C23—Br2	118.8 (4)	H54A—C54—H54C	109.5
C23—C24—C19	119.4 (6)	H54B—C54—H54C	109.5
C23—C24—H24	120.3		

### Hydrogen-bond geometry (Å, °)

**Table:**

*D*—H···*A*	*D*—H	H···*A*	*D*···*A*	*D*—H···*A*
N3—H3···O8^i^	0.90 (1)	2.73 (4)	3.453 (8)	138 (5)
N9—H9···O2	0.89 (1)	2.16 (2)	3.048 (6)	171 (6)
N6—H6···O5^ii^	0.90 (1)	2.28 (2)	3.159 (6)	165 (5)
N2—H2···O9^iii^	0.90 (1)	2.01 (2)	2.893 (6)	168 (6)
N5—H5···O6^iv^	0.90 (1)	1.99 (1)	2.885 (6)	174 (5)
N8—H8···O3^v^	0.90 (1)	1.89 (1)	2.795 (6)	179 (6)
O7—H7···N7	0.82	1.90	2.615 (6)	146.
O4—H4···N4	0.82	1.92	2.634 (6)	145.
O1—H1···N1	0.82	1.91	2.610 (6)	143.

Symmetry codes: (i) *x*, *y*+1, *z*; (ii) −*x*, *y*+1/2, −*z*+1/2; (iii) *x*, −*y*+3/2, *z*−1/2; (iv) −*x*, −*y*+1, −*z*+1; (v) *x*, −*y*+3/2, *z*+1/2.
